# Histopathological and immunohistochemical analysis of a suspected extraskeletal osteosarcoma in a rabbit (*Oryctolagus cuniculus*)

**DOI:** 10.29374/2527-2179.bjvm003324

**Published:** 2024-10-07

**Authors:** Julia Penna de Andrade, Camilla Faria Soares, André Luiz de Moura Junqueira, Daniel Reis Santos, Marcelo Pires Nogueira de Carvalho, Karen Yumi Ribeiro Nakagaki, Érica Almeida Viscone, Lize Borges, Geovanni Dantas Cassali, Rodrigo dos Santos Horta

**Affiliations:** 1 Veterinarian, Resident. Programa de Residência em Saúde Única com Ênfase em Interface Saúde Humana e Silvestre- Escola de Veterinária da Universidade Federal de Minas Gerais (UFMG). Pampulha, Belo Horizonte, MG, Brazil.; 2 Undergraduate in Veterinary Medicine, UFMG. Pampulha, Belo Horizonte, MG, Brazil.; 3 Veterinarian, DSc. Departamento de Clínica e Cirurgia Veterinária (DCCV), UFMG. Pampulha, Belo Horizonte, MG, Brazil.; 4 Veterinarian, autonomous, Celulavet - Centro de Diagnóstico Veterinário, Belo Horizonte, MG, Brazil.; 5 Veterinarian. Laboratório de Patologia Comparada, Instituto de Ciências Biológicas, UFMG. Pampulha, Belo Horizonte, MG, Brazil.

**Keywords:** non-conventional pets, oncology, osteosarcoma, pathology, immunohistochemistry, vimentin, animais de estimação não convencionais, oncologia, osteossarcoma, patologia, imuno-histoquímica, vimentina

## Abstract

An 8-year-old male rabbit (*Oryctolagus cuniculus*) presented with a subcutaneous mass in the proximal region of the fourth and accessory digit measuring 5.5 x 3.5 x 5.2cm. The mass was non-alopecic and exhibited irregular surface, ulceration and necrosis with predominantly pale and light brown coloring. Radiography revealed no involvement of bone and adjacent periosteum. The mass was marginally resected and the electrochemotherapy (ECT) was performed on the surgical bed. Histopathology and immunohistochemical analysis revealed positive reactions for Vimentin, Runx-2 and ki-67, leading to a diagnosis of extraskeletal osteosarcoma (ESOS). This report described a case of ESOS in a rabbit, thereby delineating its clinical presentation, anatomopathological characteristics, diagnostic modalities and recommended therapeutic interventions.

## Resumo

Um coelho macho de 8 anos (*Oryctolagus cuniculus*) apresentava uma massa subcutânea na região proximal do quarto e acessório dígitos medindo 5,5 x 3,5 x 5,2 cm. A massa não era alopécica e exibia superfície irregular, ulceração e necrose com coloração predominantemente pálida e marrom clara. A radiografia não revelou envolvimento de tecido ósseo ou periósteo adjacente. A massa foi submetida à excisão marginal e foi realizada eletroquimioterapia do leito cirúrgico. Histopatologia e análise imuno-histoquímica revelaram reações positivas para vimentina, Runx-2 e ki-67, levando ao diagnóstico de osteossarcoma extraesquelético. Este relato descreve um caso de osteossarcoma extraesquelético em um coelho, delineando sua apresentação clínica, características anatomopatológicas, modalidades diagnósticas e intervenções terapêuticas recomendadas.

## Introduction

Rabbits (*Oryctolagus cuniculus*) belonging to the order Lagomorpha and family Leporidae, are extensively bred for diverse purposes, including companionship, meat production and utilization in scientific research (Schoch et al., 2020). Similar to domestic animals, rabbits face an elevated risk of neoplastic diseases attributed to prolonged life expectancy (Zeeland, 2017).

Osteosarcoma is a primary bone sarcoma affecting several species, including rabbits (Fan & Khanna, 2015). Productive osteoblasic osteosarcoma is the most common bone tumor in rabbits. Although the correlation between the development of the sarcoma and body size remains unclear, age seems to be an important factor (Ishikawa et al., 2012; Silveira et al., 2016). On the other hand, extraskeletal osteosarcomas (ESOS) lack osseous tissue and involvement of adjacent periosteum and may manifest in the subcutaneous tissue or any body organ (Fan & Khanna, 2015; Hohenhaus et al., 2016; Makishima et al., 2020; Patnaik, 1990; Park et al., 2001; Renfrew et al., 2001; Wijesundera et al., 2013). Male dogs seem to be predisposed to ESOS, with primary occurrences in the spleen, liver, and kidney often metastasizing to lymph nodes and liver (Duffy et al., 2017). ESOS have been documented in three rabbits, all males over seven years old, with tumors localized on the digits of the thoracic limb, eye, and upper lips (Renfrew et al., 2001; Wijesundera et al., 2013; Makishima et al., 2020). Tumor sizes ranged from 3.5 and 4cm, with immunohistochemical analyses conducted in two cases. While no metastasis was reported, local recurrence was described in two cases.

## Case description

This paper presents a case of osteosarcoma in an eight-year-old male rabbit (*Oryctolagus cuniculus*) weighing 2.4 kg, fed with a diet of commercial bunny pellets. The rabbit resided indoors with unrestricted access to the house. Physical examination revealed bilateral cataract, onychogryphosis, and a regular body condition, with normal vital parameters. The rabbit presented a subcutaneous mass located proximally on the fourth and accessory digit, measuring 5.5 x 3.5 x 5.2cm, non-alopecic and exhibiting irregular surface, ulceration and necrosis with predominantly pale and light brown coloring. The mass had a soft to firm consistency, emitted a foul odor, and purulent discharge. The owners reported that they first noticed the mass approximately one month ago. Since then, the mass exhibited rapid and exponential growth. The animal had never been to a veterinarian prior to this consultation.

Blood samples were collected for complete blood count and biochemical analysis, while cytology was performed via fine-needle aspiration (FNA), and staging imaging tests were conducted with abdominal ultrasound, three-view chest and limb radiographs.

The erythrocyte counts and platelet exhibited no significant alterations, while the leukogram revealed leukocytosis (14.700x10^3^/uL; Ref: 4.1-10.8x10^3^/uL) attributed to heterophilia (11.025x10^3^/uL; Ref: 2.2-6.8x10^3^/uL), eosinophilia (0.137x10^3^/uL; Ref: 0.09-0.11x10^3^/uL), and mild lymphopenia (588x10^3^/uL; Ref: 15-43x10^3^/uL) (Carpenter & Harms, 2022). Serum biochemistry analysis did not reveal significant deviations, except for mild hypoproteinemia (5.63 g/dL; Ref: 6.1-7.7g/dL) and hypercalcemia (13.9 mg/dL; Ref: 7.6-12.2mg/dL) (Carpenter & Harms, 2022). Cytological examination demonstrated a red blood cell to nucleated cell ratio of less than 100, with heterophils predominating, followed by lymphocytes and macrophages. Rare to discrete multinucleated cells suggestive of osteoclasts were observed. The cytological diagnosis was inconclusive, indicating a potential heterophilic inflammatory process.

**Figure 1:**
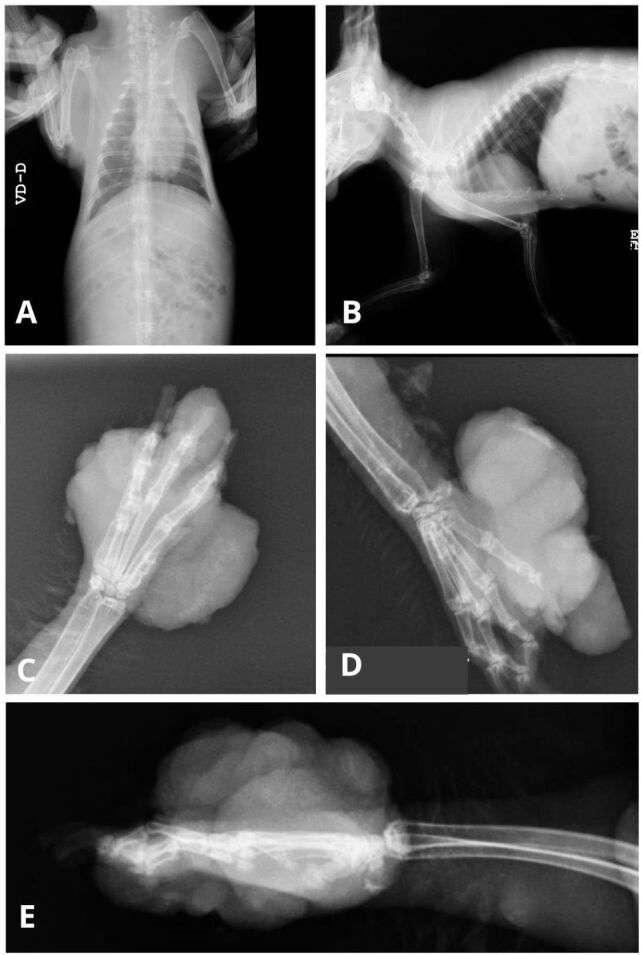
Radiographic images of an eight-year old domestic rabbit (*Oryctolagus cuniculus*) diagnosed with extraskeletal osteosarcoma. **(A)** Left laterolateral (LL) projection demonstrating normal radiographic characteristics. **(B)** Ventro-dorsal (VD) projection revealing no evidence of pulmonary opacification. **(C)** Dorsopalmar (DP) projection exhibiting irregularity and increased radiopacity. **(D)** Laterolateral (LL) projection showing increased radiopacity of soft tissues and areas of mineralization. **(E)** Mediolateral (ML) projection displaying increased radiopacity of soft tissues.

Abdominal ultrasound and chest radiographs were unremarkable (Figures 1A and 1B). Radiographs of the left thoracic limb revealed soft tissue enlargement and irregularity, alongside areas of mineralization extending from the radiocarpal joint to the distal and medium phalangeal regions of the fourth and accessory digit (Figure 1C, 1D, 1E).

The animal was then conducted for surgery. Anesthesia was induced with Diazepam (0,5mg/kg IM), Ketamine (20mg/kg IM), and Morphine (0,5mg/kg IM) and was maintained for anesthesia (trans-surgically) with Sevoflurane (2%).

**Figure 2:**
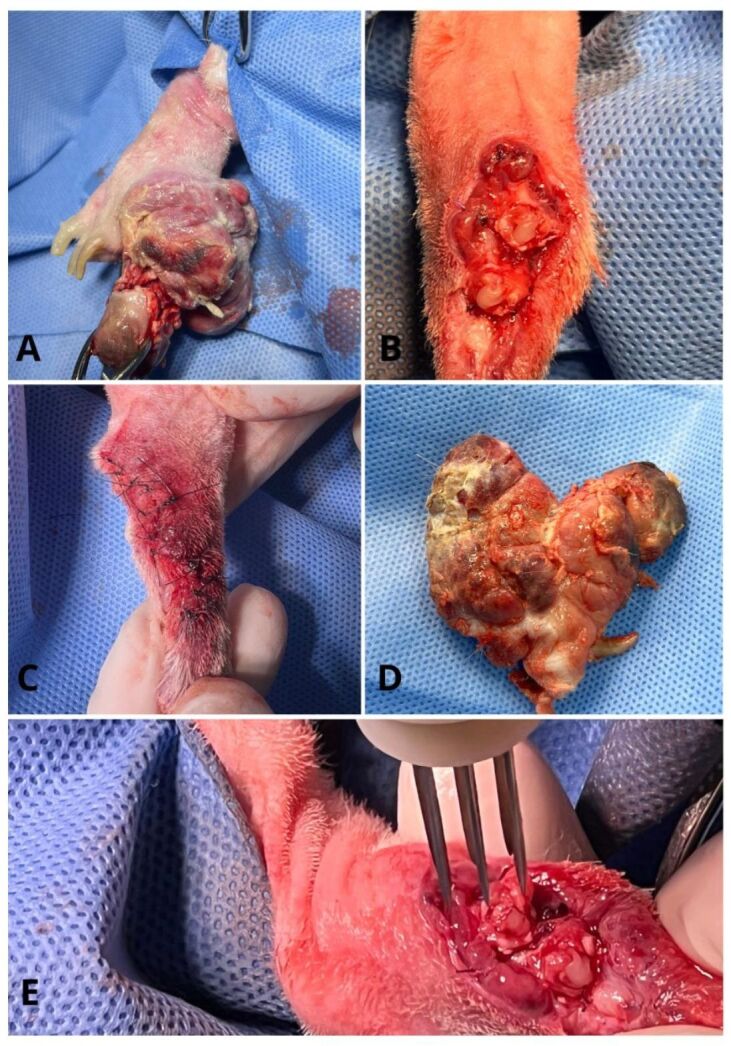
Surgical procedure depicting tumor excision and amputation of the fourth and accessory digit in an eight-year old rabbit (*Oryctolagus cuniculus*). **(A)** Surgical field preparation using Backaus forceps. **(B)** Vessel ligation conducted with 3-0 poliglecaprone 25 for excision of the neoplastic mass. **(C)** Dermorrhaphy performed with simple 5-0 nylon suture. **(D)** Excised neoplastic mass measuring 5.5 x 3.5 x 5.2 centimeters from the left thoracic. **(E)** Electrochemotherapy administration in the left thoracic limb.

Antisepsis was conducted using 2% degerming chlorhexidine and 0.5% alcoholic chlorhexidine, with the surgical field prepared using Backaus forceps (Figure 2A). Dieresis was performed through an inverted Y incision at the proximal end of the metacarpal-phalangeal joint, extending from the fourth digit to the accessory digit. Transverse sectioning of tendons, vessel ligation, and incision of the joint capsule of the proximal phalanx were carried out using an nº11 scalpel blade. Hemostasis was obtained utilizing a tourniquet applied to the radius and ulna region until the digit was excised, followed by vessel ligation using 3-0 poliglecaprone 25, compression with gauze and the application of hemostatic forceps (Figure 2B). Subcutaneous closure was achieved using poliglecaprone 3-0 and Sultan sutures, while dermorrhaphy was performed with a simple suture separated with 5-0 mononylon (Figure 2C). The excised neoplasm was sent for histopathology and immunohistochemical analysis to confirm the diagnosis (Figure 2D).

Local electrochemotherapy was conducted using bleomycin sulfate, an antitumor antibiotic utilized in therapeutic protocols for neoplasms in dogs and cats. This drug exerts cytotoxic effects that are potentiated by the regional application of electrical pulses ^(^Silveira et al., 2016). Bleomycin was administered intravenously at a dose of 15 IU/m^2^ (Castro et al., 2019). Subsequently, electroporation was initiated using the E-Pore device, calibrated for 8 pulses of 900V/cm, at 100 mS and 5000Hz, for approximately 10 minutes (with a time limit of 20 minutes) (Figure 2E).

Histopathological analysis was characterized by neoplastic cell proliferation surrounding a mineralized cartilaginous and bone matrix. The neoplastic cells exhibited marked pleomorphism, anisokaryosis, and anisocytosis, with slightly eosinophilic cytoplasm. Their nuclei ranged from rounded to elongated, with hyperchromatism and evident nucleoli. Thirteen mitotic figures were observed within 10 high-power fields (400×/2.37mm^2^). The specimen displayed lymphoplasmacytic and heterophilic inflammatory infiltrates, extensive necrotic and hemorrhagic regions, and abundant granulation tissue and cellular debris. Surgical margins were compromised, leading to a diagnosis of malignant mesenchymal neoplasia (sarcoma), with a strong indication favoring osteosarcoma, possible ESOS.

**Figure 3:**
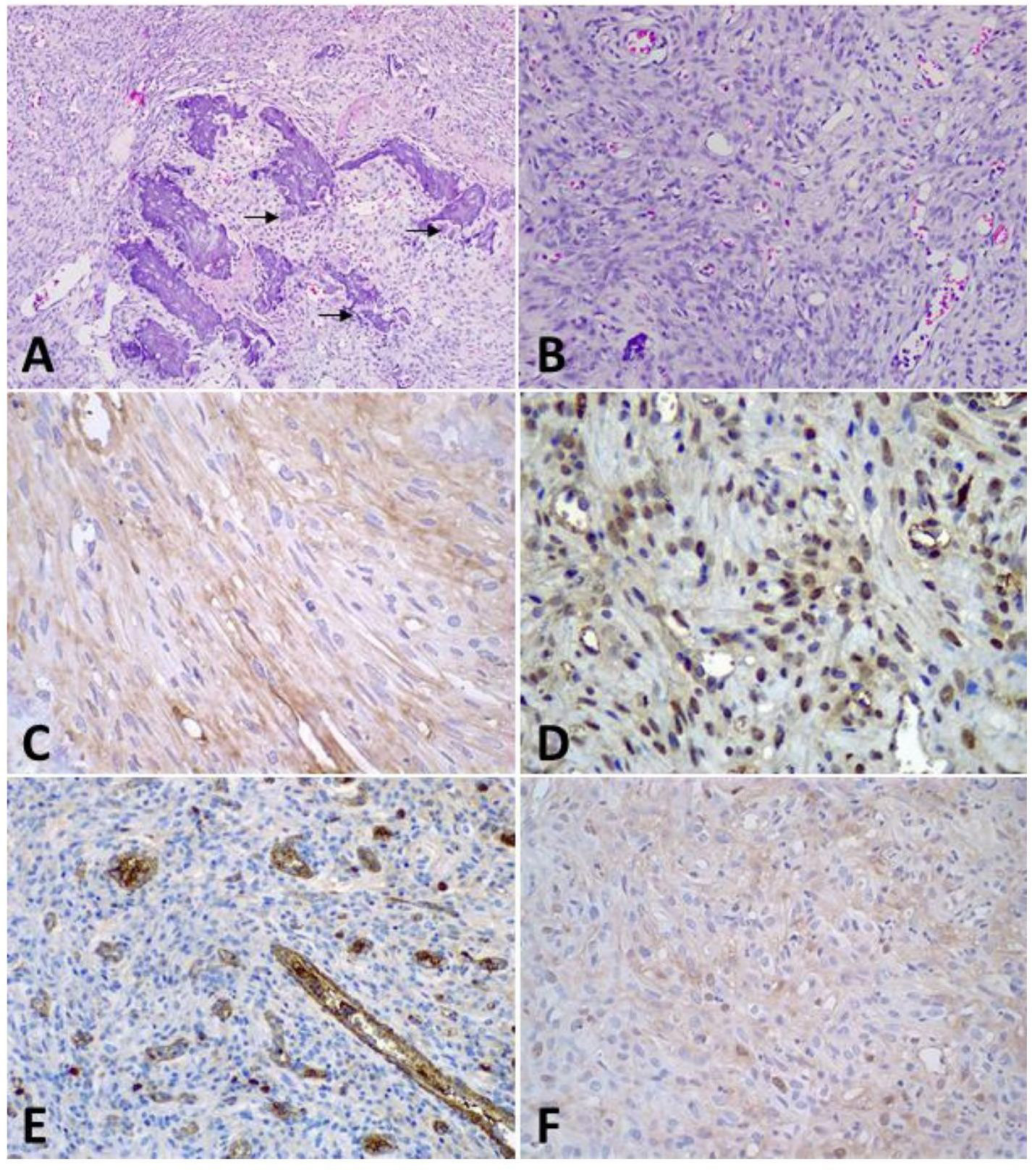
Histopathology and immunohistochemistry of extraskeletal osteosarcoma (ESOS) in an eight-year old domestic rabbit (*Oryctolagus cuniculi*). **(A)** Proliferation of neoplastic cells arranged in bundles associated with mineralized bone matrix and presence of multinucleated cells (osteoclast) (black arrow) with neovascularization. HE, 100x. **(B)** Neoplastic cells pleomorphism, cytoplasmic eosinophilia and hyperchromatosis of the cell nucleus. HE 200x. **(C)** Weak positive cytoplasmic expression of Vimentin in neoplastic cells. 600x. **(D)** Positive nuclear expression for Runx-2 (clone C-12) in neoplastic cells. 400x. **(E)** Negative cytoplasmic expression for MSM. 200x. **(F)** Positive nuclear expression for Ki67 in neoplastic cells. 400x.

Immunohistochemical analysis revealed negative immunolabeling for αSMA, MSA, and S-100. Neoplastic and multinucleated cells exhibited positive reactions for Vimentin (Figure 3C) and Runx-2 (Figure 3D), along with 8% nuclear immunolabeling for ki-67 (Figure 3F). These findings were consistent with ESOS. The definitive diagnosis was established based on the clinical presentation of a subcutaneous mass without bone or periosteum involvement, presence of neoplastic cells in bundles associated with mineralized bone matrix and the presence of osteoclast, in conjunction with positive reactions to Vimentin (Vim3B4) and Runx-2 (C-12; F-2).

The surgery and post-operative care were successful, and the animal was hospitalized for 48 hours. Despite initial signs of recovery, progressive weight loss occurred due to inadequate food supply from its owners. The animal patient was readmitted for seven days, after one week of the surgery, with no significant improvement observed, leading to the decision for euthanasia by the owners. Autopsy was not authorized.

## Discussion

Neoplasms are common in rabbits, but diagnostic, prognostic, and therapeutic criteria are still lacking. The incidence increases from 1.4% to 8.4% after the second year of life (Manning et al., 1994). In this case, the rabbit was eight years old, nearing the species' life expectancy limit, similar to the observations in middle-aged to elderly dogs and cats where neoplasm manifests in subcutaneous tissue and skin (Silveira et al., 2016). Sarcomas represent 9 to 15% of skin and subcutaneous tumors in dogs, typically presenting as solitary masses with poorly defined margins, soft and firm pseudocapsule, with infiltrative margins and susceptibility to hypo-oxygenation (Silveira et al., 2012). While there are limited reports on ESOS in rabbits, it is suggested that the evolution and biological behavior resemble those observed in other mammals, such dogs and cats (Von Bomhard et al., 2007). The mass exhibited mesenchymal origin, soft pseudocapsule and poorly defined margins, displaying progressive growth over a month along with areas of ulceration and necrosis. While the metastatic rate of soft tissue sarcomas is relatively low, ESOS metastasis is frequently observed, particularly in the liver and lymph nodes (Patnaik, 1990; Thompson & Pool, 2002). ESOS may present a more favorable prognosis when surgery is combined with local and systemic adjuvant therapies (Longhi et al., 2017), but local recurrence and distant metastasis remain the leading causes of mortality (Renfrew et al., 2001; Wijesundera et al., 2013). Visceral ESOS exhibit a more aggressive behavior, with dogs subjected to multimodal therapy experiencing mortality rates of 85-90% within two years of diagnosis (Fan & Khanna, 2015).

In rabbits, ESOS have been documented in the eye, oral cavity, and skin as white masses ranging from 2.5-35 cm in diameter, exhibiting firm to hard consistency and occasionally accompanied by multifocal areas of necrosis and hemorrhage (Wijesundera et al., 2013; Makishima et al., 2020)

An eight-year-old rabbit was diagnosed with an intraocular ESOS accompanied by pulmonary masses measuring 1cm in diameter, ultimately diagnosed as neuroendocrine carcinoma. Interestingly, there was no association between the primary intraocular neoplasm and the pulmonary masses (Makishima et al., 2020). Another study reported tumor recurrence two months after complete surgical resection, though specific details regarding surgical margins and adjuvant treatments were not provided (Wijesundera et al., 2013).

In the present case report, the leukocytosis characterized by heterophilia and eosinophilia, along with lymphopenia, may indicate an underlying inflammatory or infectious process concomitant with the neoplastic disease (Paula & Bonorino, 2023). Hypoproteinemia and hypercalcemia could be attributed to the animal's inadequate diet or disruptions in calcium metabolism, while hypercalcemia might be potentially linked to paraneoplastic syndrome, as previously documented in rabbits (Rosenthal et al., 1995; Zeeland, 2017). In this case, the rabbit exhibited an unbalanced diet, which may be a contributing factor to the observed hypercalcemia. Cytology was inconclusive but the presence of local tissue inflammation, compounded by areas of necrosis and limited cellular exfoliation, poses challenges for accurate cytological interpretation (Withrow & Macewen, 2020).

Considering the rabbit´s advanced age and likelihood of malignancy, a treatment strategy was selected. Treatment aimed at preserving quality of life and an intentional marginal excision was chosen over limb amputation. As expected, surgical margins were found to be compromised but adjuvant electrochemotherapy was anticipated for local control due to the risk of malignancy and local recurrence (Castro et al., 2019). Electrochemotherapy was performed as previously described with 8 pulses of 100mS at 900V/cm, for inducing reversible electroporation (Sloboda & Faria Júnior, 2022). Intravenous administration of Bleomycin preceded electroporation by 8 minutes, facilitating membrane destabilization and pore formation, thereby enhancing the cytotoxicity of bleomycin within neoplastic cells. This combined approach is essential, as Bleomycin's efficacy relies on its permeability into cells, necessitating its coupling with high-intensity electrical stimulation (Miklavčič et al., 2014).

In a follow-up study involving 25 cases of ESOS in humans, Vimentin positivity was observed in all tumors, with 17 demonstrating immunoreactivity for αSMA and five for S-100 antibodies (Jensen et al., 1998). Vimentin, a prominent intermediate-filament cytoplasmic protein, plays a pivotal role in mesenchymal cell cytoskeleton organization (Kuburich et al., 2022). Runx-2 serves as a primary transcription factor for endochondral and intramembranous bone formation, predominantly found in cartilaginous and bony tissues (Thompson & Pool, 2002; Wijesundera et al., 2013). The positive immunostaining for Vimentin and Runx-2 supports the mesenchymal origin of the cells and osteoblasts. While previous literature has reported the use of anti-Vimentin monoclonal antibodies and other markers for diagnosing osteosarcoma in rabbits, this study marks the first utilization of Runx-2, a crucial transcription factor in cartilaginous and bone cells, as a target for immunolabeling and diagnosis (Thompson & Pool, 2002; Wijesundera et al., 2013). Negative immunolabeling for αSMA and S-100 excludes the presence of myogenic or neurogenic cells, thereby ruling out a diagnosis of soft tissue sarcoma (Sloboda & Faria Júnior, 2022). Ki-67 is a nuclear protein expressed during all phases of the cell-cycle except G0. On the present report, the tumor exhibited an immunolabeling of 8% for Ki-67, indicative of a low growth fraction, although defined cut-offs for ESOS in rabbits are lacking. Higher expression of Ki-67 is typically associated with a poorer prognosis (Ishikawa et al., 2012).

## Conclusion

The clinical, anatomopathological, and immunohistochemical findings collectively support the diagnosis of ESO in an eight-year-old rabbit. Given the advanced age of the patient, along with the presence of locally advanced disease, aggressive behavior, and high likelihood of recurrence and metastasis, the prognosis was considered poor, but marginal resection was associated with electrochemotherapy in order to maintain quality of life and improve local disease control.
